# Mother‐child dyads living with HIV in the Western Cape, South Africa: Undetectable = Undetectable?

**DOI:** 10.1002/jia2.26418

**Published:** 2025-01-30

**Authors:** Kim Anderson, Helena Rabie, Brian S. Eley, Lisa Frigati, James Nuttall, Emma Kalk, Alexa Heekes, Gayathri Sridhar, Leigh Ragone, Vani Vannappagari, Vanessa Mudaly, Andrew Boulle, Mary‐Ann Davies

**Affiliations:** ^1^ Centre for Integrated Data and Epidemiological Research, School of Public Health, Faculty of Health Sciences, University of Cape Town Cape Town South Africa; ^2^ Department of Paediatrics and Child Health Stellenbosch University and Tygerberg Hospital Cape Town South Africa; ^3^ Paediatric Infectious Diseases Unit Department of Paediatrics and Child Health Red Cross War Memorial Children's Hospital University of Cape Town Cape Town South Africa; ^4^ Health Intelligence, Western Cape Department of Health and Wellness Cape Town South Africa; ^5^ ViiV Healthcare Durham North Carolina USA; ^6^ Western Cape Department of Health and Wellness Service Priorities Coordination Cape Town South Africa; ^7^ Division of Public Health Medicine School of Public Health, Faculty of Health Sciences, University of Cape Town Cape Town South Africa

**Keywords:** infant, child, HIV acquisitions, vertical transmission, antiretroviral therapy, viral suppression

## Abstract

**Introduction:**

Globally, children living with HIV continue to lag behind UNAIDS targets for viral suppression (VS). Because studies with linked mother‐child data are limited, we describe VS and associated factors among young children in a setting with early infant HIV testing (at birth, age 10 weeks and 6 months) and early protease inhibitor‐based first‐line antiretroviral therapy (ART).

**Methods:**

We analysed routinely collected mother‐child data for children living with HIV born 2018–2022 in Western Cape province, South Africa (followed through mid‐2023). We assessed associations between child and maternal viral load (VL) results at 12 and 24 months after child ART start using logistic regression, adjusted for child sex, birthyear, severity of child immunodeficiency at ART start, maternal age and timing of maternal HIV diagnosis.

**Results:**

Among 2219 children living with HIV; 30% were diagnosed at birth (≤7 days), 41% before age 1 year (8−365 days) and 29% at age >1 year. Overall, 5% (*n* = 112/2219) of children died, a third of whom had not started ART; 90% of children (*n* = 1990) started ART, at median age 5 months (IQR 1–16). Median follow‐up from ART start was 26 months (IQR 14–40). Among children with available VL at 12 months (*n* = 853/1582), 24 months (*n* = 614/1129) and 36 months (*n* = 350/658) after ART start, 36%, 43% and 48% were virally suppressed, respectively (VL<100 copies/ml). VS among children at 12 and 24 months was more likely if maternal VL was <100 versus ≥100 copies/ml at 12 months (adjusted odds ratio [aOR] = 3.5; 95% CI 1.9−6.5) and 24 months (aOR = 6.1; 95% CI 2.8−13.1) after child ART start. Children with no/mild versus advanced/severe immunodeficiency at ART start were more likely to achieve VS at 12 months (aOR = 2.3; 95% CI 1.3−4.2) but not at 24 months. Eligible children with missing VL at 24 months (39%) were more likely to have gaps in care of >6 months than those with VL≥100 or VL<100 copies/ml (84% vs. 28% vs. 14%, respectively; *p*<0.001).

**Conclusions:**

Less than half of children on ART achieved VS, and children were more likely to achieve VS if their mothers were also virally suppressed. Significant efforts are needed to support mother‐child dyads to achieve optimal VS.

## 1 INTRODUCTION

Due to vertical transmission prevention programmes, tremendous progress has been made in reducing the number of children acquiring HIV. However, despite these efforts, an estimated 1.4 million children aged 0–14 years are living with HIV globally, and around 120,000 children acquire HIV annually [1]. South Africa, with the largest HIV epidemic and treatment programme globally, is home to an estimated 160,000 children living with HIV (CLHIV) aged 0–14 years, with around 6500 children acquiring HIV annually [1]. Globally, CLHIV continue to fare worse than adults, including lagging well behind UNAIDS targets for viral suppression (VS). In 2023, only 48% of CLHIV aged 0–14 years had suppressed viral loads (VLs), compared to 73% of adults [2]. Particularly concerning are very young CLHIV, aged <5 years, who achieve the poorest rates of VS [3, 4]. Data on linked maternal/caregiver‐related factors are scarce but studies indicate that children are more likely to achieve VS when they and their mothers/caregivers have support from families, communities and healthcare workers [4]. In this study of a provincial birth cohort of CLHIV in South Africa, we describe characteristics and early outcomes of young children diagnosed with HIV, including VS and retention in care. To help inform interventions aimed at improving paediatric HIV outcomes, we additionally examine factors associated with children achieving VS, including maternal VS.

## 2 METHODS

Data extraction was facilitated by the Western Cape Provincial Health Data Centre (PHDC), a health information system which links individuals via unique health identifiers to a range of routine healthcare data, including outpatient visits, pharmacy records, laboratory results and hospitalizations [5, 6]. Based on these integrated data, health conditions, including HIV, are inferred for individuals. At birth, unique identifiers that are linked to maternal identifiers are issued to infants, enabling mother‐child data linkage. We included children born during a 4.5‐year period (1 May 2018−31 October 2022), living in the Western Cape province, South Africa, with PHDC‐based electronic evidence of HIV diagnosis. Extraction of a de‐identified, linked dataset was performed mid‐June 2023 (study closure), enabling follow‐up of children from birth ranging from 7 to 62 months. The study was approved by the Western Cape Department of Health Provincial Health Research Committee and the Human Research Ethics Committee at the University of Cape Town (HREC REF 145/2021).

### 2.1 Infant guidelines and definitions

We assessed CLHIV diagnosed at birth (≤7 days), 10 weeks (8−98 days) and >3 months (>98 days), as proxies for intrauterine, intrapartum/early breastfeeding and late breastfeeding transmission, respectively. These time periods coincide with routine infant HIV testing. Since 2016, infants with HIV exposure have received routine HIV‐PCR tests at birth and 10 weeks [7]. At ages 9 and 18 months, HIV‐exposed infants received HIV‐Rapid antibody testing, and testing at 6 weeks after last breastfeeding was recommended. Initial positive HIV results (HIV‐PCRs/Rapid tests) in children age <18 months were confirmed with an HIV‐PCR test. Testing guidelines changed in early 2020: birth and 10‐week HIV‐PCR testing remained but 9‐month HIV‐Rapid testing stopped [8]. Instead, 6‐month HIV‐PCR testing was introduced, and all children not known with HIV were to receive universal HIV‐Rapid testing at age 18 months, regardless of exposure status. In our analysis, in the absence of positive HIV‐PCR data, we inferred child HIV acquisition if there was alternative laboratory evidence (detectable VL at any age or positive HIV‐antigen/antibody/Rapid test at age ≥18 months) and/or triple antiretroviral therapy (ART) dispensed repeatedly, without subsequent negative HIV‐PCR/antigen/antibody tests (Table S1).

Throughout the study period, the policy in South Africa was universal ART for all young CLHIV, regardless of clinical or immunological status, with fast tracking of ART initiation (within 7 days of diagnosis). Prior to ART start, infants may have received antiretroviral drugs in accordance with national guidelines for vertical transmission prevention: daily nevirapine (NVP) for all infants with known HIV exposure; plus zidovudine (ZDV) for infants with high transmission risk [9]. The standardized first‐line ART regimen for children age ≥4 weeks and <3 years throughout the study period consisted of abacavir (ABC), lamivudine (3TC) and lopinavir‐ritonavir (LPV/r) [9]. The standardized first‐line regimen for neonates (<4 weeks old), with ART start recommended on the same day as diagnosis, comprised ZDV, 3TC and NVP [7]. After 1 month of treatment, NVP was switched to LPV/r and ZDV was switched to ABC. For children initiating ART at age ≥3 years (or ≥10 kg), ABC, 3TC + efavirenz (EFV) was recommended if not exposed to NVP for vertical transmission prevention; if exposed to NVP previously, then ABC, 3TC and LPV/r was recommended. From 2019 onwards, starting or switching to dolutegravir (DTG) tablets was possible once children weighed ≥20 kg, usually at around age 5–6 years.

Guidelines for monitoring infants and children age <5 years included CD4% at baseline, month 12 on ART and annually thereafter; VL at months 4 and 12 after ART start, and 6‐monthly thereafter. Adults on ART should have VL tests at months 4, 12 and then annually, but during breastfeeding, 6‐monthly VL tests are recommended [8]. Resistance testing was recommended if children on protease inhibitor (PI)‐based regimens for at least 2 years had >2 VL>1000 copies/ml [8]. Integrase strand transfer inhibitor resistance testing was not included with standard resistance testing. In our setting, tests that report VL<25 copies/ml as lower than the detectable limit are mostly used but if the sample volume is insufficient for this threshold of detection, then <50 or <100 is sometimes reported by laboratories as undetectable. We, therefore, assigned VL<100 copies/ml as the threshold for VS. CD4% within 1 month before/after ART start was considered as the child ART initiation measurement (baseline). For 4‐, 12‐, 24‐, 36‐ and 48‐month post‐ART outcomes (VL/CD4%), we used measurements taken within a window of 3 to <9, 9 to <18, 18 to <30, 30 to <42 and 42 to <54 months after ART start, respectively. In cases where multiple measurements were available per window, the measurement closest to the outcome date was used. We categorized child and maternal immunodeficiency categories using age‐appropriate CD4% and CD4 cell count thresholds [10].

We calculated gaps in care after ART start of >6 months between consecutive child healthcare encounters as a proxy for attrition (vs. retention) in HIV care. To calculate “gaps by 1 year” after ART start, we restricted to children with >6 months of follow‐up time since ART start and gaps starting before 18 months post‐ART start. To calculate “gaps by 2 years” after ART start, we restricted to children with >18 months of follow‐up time since ART start and gaps starting before 30 months post‐ART start.

### 2.2 Data analysis

We described characteristics of mother‐child pairs using counts and proportions, or medians with interquartile ranges, as appropriate. We used chi‐squared tests to compare proportions. We used logistic regression to assess associations between child and maternal VL results at 12 and 24 months after child ART start, adjusted for child sex, birthyear, severity of child immunodeficiency at ART start, maternal age at delivery, timing of maternal HIV diagnosis (before/during/after pregnancy) and clustering at maternal level (as some mothers had >1 child included in analyses). Because the outcome event (child VS) was not rare, we also calculated risk ratios using a modified Poisson (sensitivity) analysis; and as a substantial proportion of mothers did not have postnatal VL testing, we performed additional sensitivity analyses using extreme assumptions about missing maternal VLs (assuming all VLs≥100 vs. all VLs<100 copies/ml).

## 3 RESULTS

**Figure 1:**
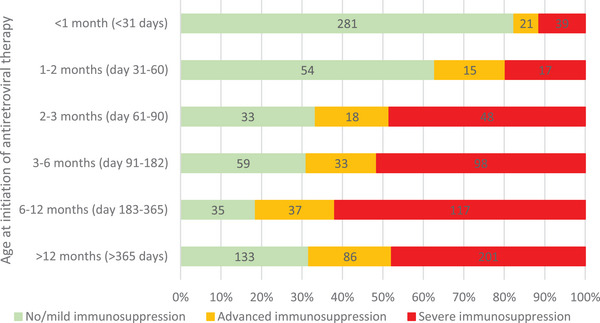
Proportion of children with varying categories of immunodeficiency at antiretroviral therapy (ART) start, by age at ART start.

**Figure 2:**
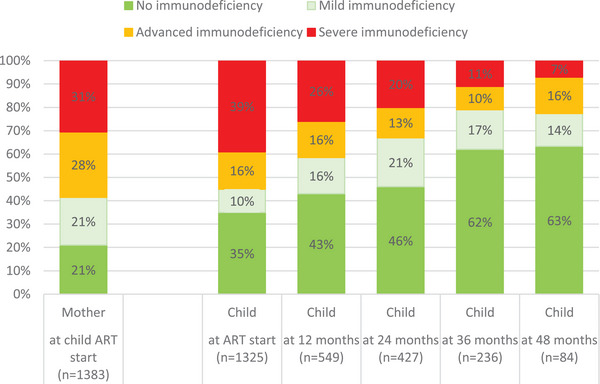
Proportion of mothers and children with varying categories of immunodeficiency at child antiretroviral therapy (ART) start, and child immunodeficiency categories at 12, 24, 36 and 48 months after ART start. *Note*: For child CD4% at ART start, an interval of 1 month (30 days) before or after ART start was used. For maternal CD4 count at child ART start, an interval of 1 year (365 days) before or after child ART start was used. For child measurements at 12, 24, 36 and 48 months after ART start, an interval of 9−18 months (274−547 days), 18−30 months (548−912 days), 30−42 months (913−1277 days) and 42−54 months (1278−1644 days) after child ART start was used. For all measurements, the one closest to the relevant timepoint was used if multiple results were available during an interval.

We included 2219 CLHIV, of whom 1275 (57%) were born to women known with HIV before or during pregnancy, 504 (23%) to mothers whose first electronic evidence of HIV was after delivery, and 440 (20%) were unlinked children (no maternal data available). Of those with linked data, 22 mothers had >1 CLHIV included (*n* = 45 CLHIV: 24 twins and 21 siblings). Of all children diagnosed with HIV, 30% were diagnosed at birth (intrauterine transmission), 18% at 10 weeks (intrapartum/early breastfeeding transmission) and 52% at >3 months (late breastfeeding transmission) (Table 1). Overall, 5% of children were recorded to have died (*n* = 112/2219), at median age 8 months (IQR 4–17), a third of whom had not started ART (*n* = 37/112). Overall, 90% of CLHIV started ART (*n* = 1990), at median age 5 months (IQR 1–16). Median time from HIV diagnosis to ART start was 13 days (IQR 6–32). Median follow‐up from birth was 38 months (IQR 24–50) and from ART start was 26 months (IQR 14–40). Older age at ART start was associated with more severe immunosuppression at ART start (Figure 1). The proportion of children with severe immunodeficiency decreased with time after ART start (Figure 2). Among CLHIV who started ART, 37% (*n* = 728/1990) started ART during hospitalization (Table 1). Among children with ≥12 months follow‐up after ART start, 31% (*n* = 479/1562) were hospitalized within the first 12 months after ART start.

**Table 1:** Characteristics of children living with HIV

Number of CLHIV (*n*=2219)	2219 (100%)
Female (*n*=2218)	1104 (49.8%)
Birthweight, grams (median, IQR) (*n*=1678)	2870 (2410−3220)
Low birthweight (<2500 g) (*n*=1678)	475 (28.3%)
Birthyear (*n*=2219)	
2018	408 (18.4%)
2019	572 (25.8%
2020	543 (24.5%)
2021	462 (20.8%)
2022	234 (10.5%)
Age at first evidence of HIV diagnosis, days (median, IQR) (*n*=2219)	107 (1−446)
Age category at first evidence of HIV diagnosis (*n*=2219)	
≤7 days	664 (29.9%)
8−98 days	408 (18.4%)
99−365 days	501 (22.6%)
366−731 days	391 (17.6%)
>731 days	255 (11.5%)
Of those with first HIV evidence at 8−98 days, CLHIV with a previous negative test (*n*=408)	
At birth (≤7 days)	157 (38.5%)
Of those with first HIV evidence at 99−365 days, CLHIV with a previous negative test (*n*=501)	
At birth only (≤7 days)	118 (23.6%)
At 8−98 days only	38 (7.6%)
Both at birth and at 8−98 days	110 (22.0%)
Of those with first HIV evidence at >365 days, CLHIV with a previous negative test (*n*=646)	
At birth only (≤7 days)	61 (9.4%)
At birth, plus at 8−98 days and/or 99−365 days	187 (28.9%)
At 8−98 days and/or 99−365 days only	56 (8.7%)
Time to ART start, among those with HIV test result available prior to ART start, days (*n*=1629) (median, IQR)	13 (6−32)
Age at ART start, days (median, IQR) (*n*=1990)	144 (32−497)
Age at ART start category (*n*=1990)	
≤7 days	243 (12.2%)
8−98 days	574 (28.8%)
99−365 days	537 (27.0%)
366−731 days	396 (19.9%)
>731 days	240 (12.1%)
Of CLHIV who started ART, those who started during a birth hospitalization (age at admission <7 days) (*n*=1990)	183 (9.2%)
Of CLHIV who started ART, those who started during a later hospitalization (age at admission ≥7 days (*n*=1990)	545 (27.4%)
CLHIV with any gap of >6 months between healthcare encounters after ART start (*n*=1804^a^)	728 (40.4%)
‐ First gap started within the first 6 months after ART start (*n*=1804^a^)	322 (17.8%)
‐ Any gap by 1 year after ART start (*n*=1804^b^)	593 (32.9%)
‐ Any gap by 2 years after ART start (*n*=1321^c^)	610 (46.2%)
‐ Returned to care before study closure (*n*=728^d^)	355 (48.8%)
‐ Gap at study closure (*n*=1772^e^)	450 (25.4%)
CLHIV with any gap at study closure of >12 months between healthcare encounters after HIV diagnosis (*n*=1722^f^)	417 (24.2%)
‐ Among those who started ART (*n*=1548^g^)	324 (20.9%)
‐ Among those who did not start ART (*n*=137^f^)	93 (67.9%)
Number of CLHIV with linked maternal data	1779 (80.2%)
Timing of maternal HIV diagnosis, per child (*n*=1779)	
Before the pregnancy	891 (50.1%)
During the pregnancy (< delivery date)	384 (21.6%)
After the pregnancy (≥ delivery date)	504 (28.3%)
Maternal age at delivery, per child, years (median, IQR) (*n*=1779)	28.1 (24.2−32.5)
Maternal parity^h^ at the time of pregnancy, per child (*n*=1779)	
0	855 (48.1%)
1	503 (28.3%)
≥2	421 (23.7%)

Abbreviations: ART, antiretroviral therapy; CLHIV, children living with HIV; IQR, interquartile range.

^a^ Restricted to: CLHIV with >6 months of follow‐up time since ART start; gap/s started after ART start.

^b^ Restricted to: CLHIV with >6 months of follow‐up time since ART start; gap/s started after ART start and started before 18 months post‐ART start.

^c^ Restricted to: CLHIV with >18 months of follow‐up time since ART start; gap/s started after ART start and started before 30 months post‐ART start.

^d^ Restricted to: CLHIV with >6 months of follow‐up time since ART start who had any gap/s starting after ART start; return excluded if returning date coincided with death date.

^e^ Restricted to CLHIV with >6 months of follow‐up time since ART start and presumed alive at study closure.

^f^ Restricted to CLHIV with >12 months of follow‐up time since HIV diagnosis and presumed alive at study closure.

^g^ Restricted to CLHIV with >12 months of follow‐up time since ART start and presumed alive at study closure.

^h^ Based on prior digital evidence of pregnancy in the Western Cape province.

Most children (96%) received LPV/r‐based regimens; very few (beyond the neonatal period) received non‐nucleoside reverse transcriptase inhibitor (NNRTI)‐based regimens. Only 20 children (1%) were prescribed DTG, of whom three were initiated on DTG as part of their starting regimen and the rest were prescribed DTG as either a single‐drug switch after receiving prior LPV/r or as part of a second‐line regimen.

**Figure 3:**
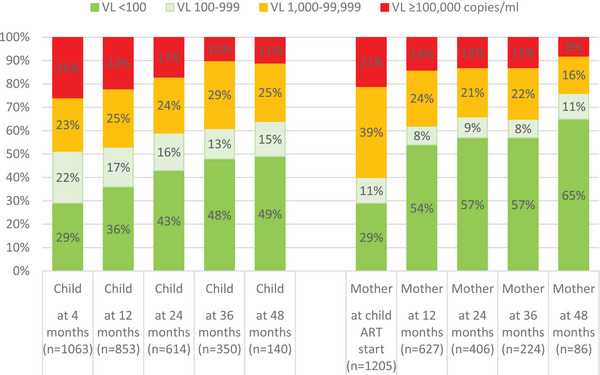
Proportion of mothers and children with varying categories of viral load (VL) at child antiretroviral therapy (ART) start and at 4, 12, 24, 36 and 48 months after child ART start. *Note*: For child measurements at 4, 12, 24, 36 and 48 months after ART start, an interval of 3−9 months (91−273 days), 9−18 months (274−547 days), 18−30 months (548−912 days), 30−42 months (913−1277 days) and 42−54 months (1278−1644 days) after child ART start was used. The same intervals were used for maternal measurements, except that for maternal VL at 12 months after child ART start, an interval of 6−18 months (182−547 days) was used. For maternal VL at child ART start, an interval of 12 months (365 days) before to 6 months (181 days) after child ART start was used. For all measurements, the one closest to the relevant timepoint was used if multiple results were available during an interval.

The proportion of eligible CLHIV with an available VL at 12, 24 and 36 months after ART start was 59%, 61% and 64%, respectively (*n* = 853/1438; *n* = 614/1001; and *n* = 350/544; children were not considered eligible for “missing VLs” unless they had a minimum of 18‐, 30‐ and 42‐months’ follow‐up post‐ART start, respectively). Among children with an available VL, 36%, 43% and 48% were virally suppressed at 12, 24 and 36 months after ART start, respectively (VL<100 copies/ml) (Figure 3). Among mothers with an available VL, 29%, 54%, 57% and 57% were virally suppressed at child ART start, 12, 24 and 36 months after child ART start, respectively (Figure 3).

Univariate analyses of factors associated with child VS are shown in Table S2. In multivariate analysis, VS among children at 12 months was more likely if the maternal VL nearest child VL measurement was <100 versus ≥100 copies/ml (adjusted odds ratio [aOR] = 3.5; 95% CI 1.9−6.5). Similarly, VS was more likely among children at 24 months if the maternal VL nearest child VL measurement was <100 versus ≥100 copies/ml (aOR = 6.1; 95% CI 2.8−13.1) (Table 2). Children with no/mild versus advanced/severe immunodeficiency at ART start were more likely to be virally suppressed at 12 months (aOR = 2.3; 95% CI 1.3−4.2) but not at 24 months. Sensitivity analyses demonstrate that maternal VS remains significantly associated with child VS, even when accounting for the common nature of the outcome and under extreme assumptions about missing maternal VL data (Tables S3−S5).

**Table 2:** Multivariate logistic regression analyses of factors associated with child viral suppression at 12 and 24 months after antiretroviral therapy start

	VS at 12 months (*n*=269)		VS at 24 months (*n*= 171)	
	aOR (95% CI)	*p*	aOR (95% CI)	*p*
Female sex (vs. male)	1.36 (0.78−2.39)	0.28	0.45 (0.22−0.91)	0.03
Birthyear				
2018	Ref		Ref	
2019	1.66 (0.72−3.82)	0.23	0.79 (0.31−2.01)	0.63
2020	1.22 (0.56−2.67)	0.62	0.90 (0.36−2.28)	0.83
2021	0.57 (0.25−1.30)	0.18	0.43 (0.08−2.37)	0.33
2022	0.16 (0.02−1.57)	0.12	(n/a)	
Child immunodeficiency category (CD4% category) at ART start				
Severe/advanced immunodeficiency	Ref		Ref	
No/mild immunodeficiency	2.34 (1.32−4.15)	0.004	1.39 (0.67−2.86)	0.37
Maternal age at delivery				
25 to <35 years	Ref		Ref	
<20 years	0.60 (0.17−2.15)	0.44	0.40 (0.08−1.96)	0.26
20 to <25 years	0.89 (0.42−1.89)	0.76	0.54 (0.21−1.39)	0.20
≥35 years	0.47 (0.21−1.05)	0.07	0.83 (0.27−2.58)	0.74
Timing of maternal HIV diagnosis				
Before the pregnancy	Ref		Ref	
During the pregnancy^a^	0.86 (0.42−1.77)	0.69	0.40 (0.16−1.01)	0.05
After the pregnancy^b^	1.88 (0.96−3.68)	0.07	0.60 (0.24−1.49)	0.27
Maternal viral load at 12 or 24 months after child ART start, respectively				
≥100 copies/ml	Ref		Ref	
<100 copies/ml	3.53 (1.94−6.41)	<0.001	6.07 (2.71−13.59)	<0.001

Abbreviations: aOR, adjusted odds ratio; ART, antiretroviral therapy; CI, confidence interval; VS, viral suppression (viral load <100 copies/ml).

^a^ Before delivery date.

^b^ On or after delivery date.

Children with missing VL at 1 year after ART start were more likely to have experienced gaps in care (of >6 months) by 1 year since ART start than those with VL≥100 or VL<100 copies/ml at 1 year (*n* = 70% vs. 16% vs. 6%, *p*<0.001; *n* = 412/585, 87/542 and 19/311, respectively). Similarly, children with missing VL at 2 years after ART start were more likely to have experienced gaps in care by 2 years since ART start than those with VL≥100 or VL<100 copies/ml at 2 years (84% vs. 28% vs. 14%, *p*<0.001; *n* = 327/387, 98/351 and 37/263, respectively). Among children who started ART, gaps in care of >6 months were common: 33% and 46% of children experienced gaps by 1 and 2 years after ART start, respectively (Table 1). Returning to care after such gaps occurred frequently; 49% of children with a gap returned to care. At study closure, 21% of children with >12 months follow‐up time since ART start and presumed alive, had no healthcare encounters for >12 months since ART start (*n* = 324/1548). Maternal deaths were recorded for 4% of children with linked maternal data (*n* = 65/1779). Median maternal age at death was 31 years (IQR 28–35) and child age at maternal death was 25 months (IQR 16–33).

Resistance testing was conducted in 38 children on ART, of whom 5 (13%), 18 (47%) and 26 (68%) had intermediate/high‐level resistance detected to PIs, NNRTIs and nucleoside reverse transcriptase inhibitors (NRTIs), respectively. Of those with NRTI resistance detected (*n* = 26), all children were resistant to 3TC, eight of whom had additional ABC resistance and one child also had ZDV resistance. Overall, 10 children in the cohort were changed to second‐line regimens.

## 4 DISCUSSION

In our setting, the majority of CLHIV are born to women known with HIV before or during pregnancy, with higher transmission rates associated with inconsistent maternal ART usage during pregnancy, low maternal CD4 count and elevated maternal VL [11]. The majority of children (52%) were diagnosed with HIV after 3 months of age, suggesting late breastfeeding transmission in most cases, although the timing of HIV acquisition was often uncertain as only 41% of children diagnosed with HIV after 3 months of age (*n* = 476/1147) had negative birth tests recorded.

The majority of newly diagnosed young CLHIV in our setting successfully linked to HIV services and initiated ART (90%). However, 2 years after ART start, only 43% of children with available VLs were virally suppressed. Furthermore, 39% of children did not have available VL, and of these, 84% had experienced a gap in care of >6 months since ART start, suggesting high levels of attrition to care among those with missing VL. Our province‐wide findings of a large proportion of CLHIV without VL recorded and less than half of young children on ART with VL available achieving VS are disappointing, and are consistent with previous national findings of only around half of children age <15 years receiving annual VL monitoring and less than half of CLHIV age <5 years being virally suppressed [3]. Despite the shift to earlier infant diagnosis and earlier ART start in South Africa from 2016, rates of VS among children have remained relatively static.

Globally, VS rates among children receiving ART have remained worse than among adults, partly due to suboptimal paediatric ART medication as well as challenges in retaining children in care [2]. To address the disparity of poor VS rates among children, outdated paediatric treatment regimens must urgently be replaced with optimized ART for all children [12]. The introduction of generic, child‐friendly DTG is a significant breakthrough that could transform paediatric outcomes. To date, it is estimated that 62% of children on ART globally are receiving DTG‐based regimens [13]. Although coverage of paediatric DTG has almost doubled since 2021, it is still far short of adult DTG coverage which is in excess of 90%. In South Africa, national ART guidelines were updated in May 2023 and ABC‐3TC‐DTG is now recommended as the preferred first‐line ART regimen for CLHIV from age ≥4 weeks and weighing ≥3 kg [14]. It is hoped that transitioning all children on ART to DTG‐based regimens may significantly improve paediatric VS rates, as DTG‐based regimens have several advantages, including better palatability and once‐daily dosing (vs. twice‐daily LPV/r dosing), more rapid VS, fewer side effects and higher genetic barrier to developing drug resistance. However, additional obstacles to child ART outcomes must be considered, beyond providing optimized ART for children.

Retaining children in care remains challenging, as reflected by the high proportion of children in our cohort with gaps in care of >6 months. Our findings of 40% overall with such gaps, including 18% with gaps starting within the first 6 months of ART start, are similar to findings of a large Southern African study of >50,000 CLHIV age <16 years initiating ART between 2004 and 2016, which found that 44% of CLHIV had gaps in care of >6 months, including 20% within the first 6 months of ART start [15]. Furthermore, the authors found that gaps in care within 6 months of ART start were associated with mortality hazards twice as high, suggesting that improving retention in care of CLHIV in the early period after ART start is crucial for improving outcomes.

For young children, ART adherence is heavily dependent on the motivation and dedication of their caregivers. In the era of universal ART, the majority of CLHIV are born to mothers who for complex social reasons, including poverty, stigma, and drug and alcohol abuse, do not access or take ART consistently, and many of these mothers, in turn, find it difficult to access or give their children ART consistently [16]. We found that mothers who achieve VS are more likely to have children who achieve VS. Similarly, a Kenyan study of 1698 CLHIV age <15 years with linked caregiver data available, found that children were at higher risk of viral non‐suppression if their caregivers were not virally suppressed (vs. caregivers with VS) [17]. This apparent association between child and mother/caregiver VL strongly suggests that children's VL should be closely monitored if their primary caregivers are not virally suppressed. Other studies in our region have found that children who start ART in early infancy and have poor viral responses have mothers who are more likely to report adverse social life events [18] and maternal ART interruptions [19]. Family‐centred approaches [20] and better attention to broader social support for the most vulnerable mothers are needed for more successful HIV prevention, care and treatment.

Among children starting ART, 27% were hospitalized at the time of ART start (excluding those who started ART during birth admissions) and 31% of CLHIV were hospitalized within a year after ART start, underscoring the burden of morbidity in this vulnerable group. These findings are similar to those in an earlier Western Cape birth cohort of 840 CLHIV who started ART at age <3 months (2013−2017), of whom 25% started ART during a hospitalization (excluding birth admissions) and 40% of whom were hospitalized within the first year after ART start [21].

A substantial proportion (24%) of all children diagnosed with HIV and presumed alive had no healthcare encounters for >12 months at study closure. While some children seemingly lost to care may return to care or may have relocated care to other provinces, it is likely that a significant number would have died. Death data were only available for children who died in medical facilities or whose deaths were reported to facilities and are, therefore, very likely under‐ascertained. Mortality in CLHIV is substantially underestimated in studies using routine data, when children who have died are misclassified as lost to care [22]. A study of 680 children and youth aged <25 years on ART who were lost and traced in Southern Africa (2017–2019) found high mortality (9%) among those considered lost, with mortality the highest for those starting ART at <2 years of age [23]. Retaining children in care who start ART very young needs to be prioritized; children who miss healthcare visits should be actively traced to ensure they return to care. Barriers to care for children and their caregivers should be identified and addressed.

### 4.1 Study limitations

Our large province‐wide sample size of young CLHIV is a major strength of our analysis. However, our cohort may not be representative of all young CLHIV in the province, or other regions. More than half of CLHIV in South Africa are estimated to have died before age 5 years without HIV being diagnosed [24], therefore, survival bias is likely present in our cohort. Conversely, among surviving 5‐year‐old CLHIV in South Africa, up to 30% may remain undiagnosed [24, 25]. Undiagnosed survivors may include “slow progressors” who only present to healthcare services in older childhood [26], in comparison to many children in our cohort who were ill and hospitalized, and, therefore, diagnosed, at an earlier age. We may have missing data because of the limitations of routine data collected in a programmatic setting. Apart from likely missing mortality data, there may be some missing pharmacy (ART) data, as most but not all facilities in the province have electronic dispensing data. Maternal and child HIV‐Rapid testing data were likely incomplete as HIV‐Rapid results are not routinely captured electronically by all facilities, therefore, timing of HIV diagnoses may be incorrectly inferred. Inter‐provincial mobility is relatively common and as the PHDC does not receive data from other provinces, we may have missing data, including pharmacy, laboratory and mortality data. We were not able to examine associations with VL at ART start as this test is not routinely performed in our setting. Although our overall CLHIV cohort size was large, because only around half the children, and even fewer mothers, had VLs recorded, only a subset of mother‐child dyads could be included in complete‐case regression analyses of associations between mother‐child VL. Additionally, we did not have data on who the children's caregivers were, and it is possible that persons other than the mothers were caregivers. At least 4% of children had alternative caregivers as their mothers had died by study closure.

## 5 CONCLUSIONS

Less than half of young children on ART achieve VS. While advocating for improved coverage with better paediatric regimens is crucial, addressing less tangible barriers, such as retention in care and adherence support, remains paramount. Children are more likely to achieve VS if their mothers are also virally suppressed. Significant efforts are needed to support mother‐child dyads to achieve optimal VS. In the era of universal maternal ART access, mothers of children with vertically acquired HIV represent a particularly vulnerable group who require enhanced adherence support and social support to both take their own ART and administer child ART consistently.

## COMPETING INTERESTS

KA, M‐AD and EK received funding from ViiV Healthcare for this project. GS, LR and VV are full‐time employees at ViiV Healthcare and have stock ownership. The other authors have no conflicts of interest to declare.

## AUTHORS’ CONTRIBUTIONS

M‐AD contributed to study conception and design; AH performed data extraction; KA performed data analysis and drafted the manuscript; HR, BSE, LF and JN provided clinical care to CLHIV in the Western Cape; all authors reviewed and approved the manuscript before submission.

## FUNDING

KA, M‐AD and EK received funding from ViiV Healthcare for this project. The PHDC was supported by grant number U01AI069924 from NIH (NIAID, NICHD, NCI, NIDA, NIMH)—PI: M. Egger and M‐A. Davies. AB, M‐AD and EK were supported by grant number R01HD080465 from NIH (NICHD).

## Supporting information



Additional file, titled “Supplementary Material,” contains supplementary tables (Tables S1−S5).

## Data Availability

The data that support the findings of this study are available on request from the corresponding author. The data are not publicly available due to privacy or ethical restrictions.
