# Supplementary material for: Mother‐child dyads living with HIV in the Western Cape, South Africa: Undetectable = Undetectable?
Source: J Int AIDS Soc. 2025 Jan 30;28(2):e26418. doi: 10.1002/jia2.26418 (PMC11782834; doi:10.1002/jia2.26418)
Supplement: Supplementary file 1 — Additional file, titled “Supplementary Material,” contains supplementary tables (Tables S1−S5). [file JIA2-28-e26418-s001.docx]

**Table S1: Electronic evidence of HIV diagnosis in 2219 children**

| Positive HIV-PCR test/s | 1790 (80.7%) |
| --- | --- |
| Alternative laboratory test/s and ART | 280 (12.6%) |
| ART only | 80 (3.6%) |
| Alternative laboratory test/s only | 69 (3.1%) |

Abbreviation: ART antiretroviral therapy

Note: Alternative laboratory tests include detectable viral load at any age or positive HIV-antigen/antibody/Rapid test at age ≥18 months

**Table S2: Univariate logistic regression analyses of factors associated with child viral suppression at 12 and 24 months after antiretroviral therapy start**

|  |  | VS at 12 months | | VS at 24 months | |
| --- | --- | --- | --- | --- | --- |
|  |  | **OR (95% CI)** | **p** | **OR (95% CI)** | **p** |
| Female sex (vs male) (n=849; 610) |  | 1.32 (1.00-1.75) | 0.05 | 0.91 (0.66-1.25) | 0.56 |
| Birthyear (n=849; 610) | 2018 | Ref |  | Ref |  |
|  | 2019 | 0.96 (0.65-1.43) | 0.85 | 0.91 (0.60-1.37) | 0.64 |
|  | 2020 | 1.01 (0.68-1.49) | 0.97 | 0.79 (0.52-1.21) | 0.28 |
|  | 2021 | 0.66 (0.42-1.03) | 0.07 | 0.87 (0.48-1.60) | 0.66 |
|  | 2022 | 0.37 (0.13-1.03) | 0.06 | n/a |  |
| Child age at ART start (n=849; 610) | ≤7 days | Ref |  | Ref |  |
|  | 8-98 days | 1.16 (0.74-1.80) | 0.52 | 0.81 (0.50-1.31) | 0.39 |
|  | 99-365 days | 0.82 (0.52-1.31) | 0.41 | 0.96 (0.59-1.59) | 0.89 |
|  | 366-731 days | 1.09 (0.66-1.79) | 0.75 | 0.79 (0.46-1.37) | 0.40 |
|  | >731 days | 1.16 (0.62-2.19) | 0.64 | 0.79 (0.30-2.09) | 0.63 |
| Child immunodeficiency category (CD4% category) at ART start | No/mild immunodeficiency | Ref |  | Ref |  |
| (n=849;610) ^a^ | Advanced immunodeficiency | 0.38 (0.21-0.67) | 0.001 | 0.89 (0.49-1.61) | 0.69 |
|  | Severe immunodeficiency | 0.64 (0.44-0.92) | 0.02 | 0.66 (0.43-1.03) | 0.07 |
|  | Unknown | 0.74 (0.52-1.04) | 0.08 | 0.84 (0.56-1.24) | 0.37 |
| Maternal immunodeficiency category (CD4 count category) nearest child ART start (n=849; 610) ^b^ | No/mild immunodeficiency | Ref |  | Ref |  |
|  | Advanced immunodeficiency | 0.81 (0.55-1.21) | 0.31 | 1.01 (0.64-1.58) | 0.98 |
|  | Severe immunodeficiency | 0.71 (0.48-1.05) | 0.08 | 0.81 (0.52-1.28) | 0.37 |
|  | Unknown | 0.84 (0.58-1.22) | 0.36 | 1.51 (0.98-2.32) | 0.06 |
| Timing of maternal HIV diagnosis, per child | Before the pregnancy | Ref |  | Ref |  |
| (n=713; 515) | During the pregnancy (< delivery date) | 1.17 (0.79-1.73) | 0.43 | 1.02 (0.66-1.57) | 0.92 |
|  | After the pregnancy (≥ delivery date) | 1.57 (1.10-2.25) | 0.01 | 0.98 (0.64-1.52) | 0.94 |
| Maternal age at delivery (n=713; 515) | 25 to <35 years | Ref |  | Ref |  |
|  | <20 years | 0.72 (0.39-1.32) | 0.29 | 0.49 (0.21-1.14) | 0.10 |
|  | 20 to <25 years | 0.72 (0.47-1.08) | 0.11 | 1.04 (0.66-1.63) | 0.88 |
|  | ≥35 years | 0.79 (0.51-1.25) | 0.32 | 1.08 (0.64-1.81) | 0.78 |
| Maternal VL at 12 or 24 months after child ART start, respectively | ≥100 copies/ml | Ref |  | Ref |  |
| (n=849; 610) ^c^ | <100 copies/ml | 2.95 (1.89-4.59) | <0.001 | 3.11 (1.84-5.24) | <0.001 |
|  | Unknown | 1.85 (1.23-2.78) | 0.003 | 1.92 (1.21-3.06) | 0.01 |

Abbreviations: VS viral suppression, OR odds ratio, CI confidence interval, ART antiretroviral therapy, VL viral load. Note: Four children with ART start at age >6 months were excluded from analyses due to undetectable VL recorded at ART start (therefore they likely were on ART prior to recorded ART start date. Per variable, (n=x; y) indicates the numbers included in univariable analysis of VS at 12 months (x) and 24 months (y) ^a^ (Unknown=274; 212) ^b^ (Unknown=213; 152) ^c^ (Unknown=454; 347)

**Table S3: Modified Poisson regression analyses (with cluster-robust standard errors) of factors associated with child viral suppression at 12 and 24 months after antiretroviral therapy start**

|  |  | VS at 12 months (n=269) | | VS at 24 months (n= 171) | |
| --- | --- | --- | --- | --- | --- |
|  |  | **aRR (95% CI)** | **p** | **aRR (95% CI)** | **p** |
| Female sex (vs male) |  | 1.19 (0.87-1.62) | 0.27 | 0.70 (0.50-0.96) | 0.03 |
| Birthyear | 2018 | Ref |  | Ref |  |
|  | 2019 | 1.32 (0.88-1.98) | 0.17 | 0.92 (0.60-1.41) | 0.70 |
|  | 2020 | 1.13 (0.75-1.69) | 0.56 | 0.95 (0.65-1.38) | 0.79 |
|  | 2021 | 0.74 (0.47-1.18) | 0.21 | 0.67 (0.29-1.59) | 0.37 |
|  | 2022 | 0.31 (0.05-1.89) | 0.20 | (n/a) |  |
| Child immunodeficiency category (CD4% category) at ART start | Severe/advanced immunodeficiency | Ref |  | Ref |  |
|  | No/mild immunodeficiency | 1.57 (1.15-2.13) | 0.004 | 1.16 (0.84-1.60) | 0.37 |
| Maternal age at delivery | 25 to <35 years | Ref |  | Ref |  |
|  | <20 years | 0.74 (0.36-1.52) | 0.42 | 0.60 (0.24-1.53) | 0.29 |
|  | 20 to <25 years | 0.94 (0.64-1.38) | 0.75 | 0.73 (0.43-1.24) | 0.25 |
|  | ≥35 years | 0.66 (0.40-1.10) | 0.11 | 0.90 (0.58-1.41) | 0.66 |
| Timing of maternal HIV diagnosis | Before the pregnancy | Ref |  | Ref |  |
|  | During the pregnancy^a^ | 0.93 (0.61-1.41) | 0.72 | 0.66 (0.43-1.03) | 0.07 |
|  | After the pregnancy^b^ | 1.35 (0.96-1.90) | 0.09 | 0.82 (0.55-1.21) | 0.31 |
| Maternal viral load at 12 or 24 months after child ART start, respectively | ≥100 copies/ml or unknown | Ref |  | Ref |  |
|  | <100 copies/ml^c^ | 2.15 (1.46-3.17) | <0.001 | 2.68 (1.66-4.32) | <0.001 |
| Abbreviations: VS viral suppression (viral load <100 copies/ml); aRR adjusted Risk Ratio; CI confidence interval, ART antiretroviral therapy  ^a^ Before delivery date; ^b^ On or after delivery date; ^c^ Composite category, combining known viral loads <100 copies/ml with unknown viral loads | | | | | |

**Table S4: Multivariate logistic regression analyses of factors associated with child viral suppression at 12 and 24 months after antiretroviral therapy start: sensitivity analyses assuming missing maternal viral loads are ≥100 copies/ml**

|  |  | VS at 12 months (n=490) | | VS at 24 months (n= 342) | |
| --- | --- | --- | --- | --- | --- |
|  |  | **aOR (95% CI)** | **p** | **aOR (95% CI)** | **p** |
| Female sex (vs male) |  | 1.33 (0.90-1.96) | 0.15 | 0.85 (0.53-1.35) | 0.49 |
| Birthyear | 2018 | Ref |  | Ref |  |
|  | 2019 | 1.07 (0.60-1.90) | 0.83 | 0.72 (0.39-1.33) | 0.30 |
|  | 2020 | 1.06 (0.62-1.83) | 0.83 | 0.79 (0.43-1.43) | 0.43 |
|  | 2021 | 0.66 (0.36-1.19) | 0.17 | 0.84 (0.38-1.87) | 0.68 |
|  | 2022 | 0.33 (0.10-1.12) | 0.08 | (n/a) |  |
| Child immunodeficiency category (CD4% category) at ART start | Severe/advanced immunodeficiency | Ref |  | Ref |  |
|  | No/mild immunodeficiency | 2.03 (1.37-3.01) | <0.001 | 1.62 (1.02-2.58) | 0.04 |
| Maternal age at delivery | 25 to <35 years | Ref |  | Ref |  |
|  | <20 years | 0.80 (0.35-1.81) | 0.59 | 0.55 (0.19-1.64) | 0.29 |
|  | 20 to <25 years | 0.79 (0.48-1.31) | 0.36 | 0.93 (0.51-1.72) | 0.82 |
|  | ≥35 years | 0.64 (0.35-1.15) | 0.13 | 1.07 (0.53-2.18) | 0.85 |
| Timing of maternal HIV diagnosis | Before the pregnancy | Ref |  | Ref |  |
|  | During the pregnancy^a^ | 0.89 (0.54-1.47) | 0.65 | 0.83 (0.46-1.47) | 0.51 |
|  | After the pregnancy^b^ | 1.74 (1.09-2.80) | 0.02 | 0.98 (0.54-1.76) | 0.94 |
| Maternal viral load at 12 or 24 months after child ART start, respectively | ≥100 copies/ml or unknown^c^ | Ref |  | Ref |  |
|  | <100 copies/ml | 1.86 (1.23-2.79) | 0.003 | 3.01 (1.82-4.99) | <0.001 |
| Abbreviations: VS viral suppression (viral load <100 copies/ml); aOR adjusted Odds Ratio; CI confidence interval, ART antiretroviral therapy  ^a^ Before delivery date; ^b^ On or after delivery date; ^c^ Composite category, combining known viral loads ≥100 copies/ml with unknown viral loads | | | | | |

**Table S5: Multivariate logistic regression analyses of factors associated with child viral suppression at 12 and 24 months after antiretroviral therapy start: sensitivity analyses assuming missing maternal viral loads are <100 copies/ml**

|  |  | VS at 12 months (n=490) | | VS at 24 months (n= 342) | |
| --- | --- | --- | --- | --- | --- |
|  |  | **aOR (95% CI)** | **p** | **aOR (95% CI)** | **p** |
| Female sex (vs male) |  | 1.30 (0.87-1.92) | 0.20 | 0.80 (0.50-1.27) | 0.34 |
| Birthyear | 2018 | Ref |  | Ref |  |
|  | 2019 | 1.12 (0.62-2.00) | 0.71 | 0.81 (0.44-1.49) | 0.50 |
|  | 2020 | 1.16 (0.67-2.01) | 0.61 | 0.87 (0.48-1.57) | 0.64 |
|  | 2021 | 0.68 (0.38-1.22) | 0.19 | 0.63 (0.29-1.37) | 0.25 |
|  | 2022 | 0.29 (0.09-0.95) | 0.04 | (n/a) |  |
| Child immunodeficiency category (CD4% category) at ART start | Severe/advanced immunodeficiency | Ref |  | Ref |  |
|  | No/mild immunodeficiency | 2.15 (1.44-3.20) | <0.001 | 1.84 (1.15-2.92) | 0.01 |
| Maternal age at delivery | 25 to <35 years | Ref |  | Ref |  |
|  | <20 years | 0.74 (0.33-1.67) | 0.47 | 0.60 (0.21-1.68) | 0.33 |
|  | 20 to <25 years | 0.74 (0.44-1.25) | 0.27 | 0.98 (0.54-1.78) | 0.95 |
|  | ≥35 years | 0.63 (0.35-1.11) | 0.11 | 1.23 (0.61-2.49) | 0.57 |
| Timing of maternal HIV diagnosis | Before the pregnancy | Ref |  | Ref |  |
|  | During the pregnancy^a^ | 0.92 (0.56-1.52) | 0.74 | 0.81 (0.46-1.42) | 0.47 |
|  | After the pregnancy^b^ | 1.85 (1.16-2.96) | 0.01 | 1.04 (0.59-1.85) | 0.89 |
| Maternal viral load at 12 or 24 months after child ART start, respectively | ≥100 copies/ml or unknown | Ref |  | Ref |  |
|  | <100 copies/ml^c^ | 2.66 (1.59-4.45) | <0.001 | 3.12 (1.66-5.86) | <0.001 |
| Abbreviations: VS viral suppression (viral load <100 copies/ml); aOR adjusted Odds Ratio; CI confidence interval, ART antiretroviral therapy  ^a^ Before delivery date; ^b^ On or after delivery date; ^c^ Composite category, combining known viral loads <100 copies/ml with unknown viral loads | | | | | |
